# 640-Slice CT Measurement of Superior Orbital Fissure as Gateway for Light into the Brain: Statistical Evaluation of Area and Distance

**DOI:** 10.1371/journal.pone.0162940

**Published:** 2016-09-23

**Authors:** Alice La Marra, Simone Quarchioni, Fabiana Ferrari, Giovanni Luca Gravina, Antonio Barile, Lorenzo Maria Gregori, Ernesto Di Cesare, Alessandra Splendiani

**Affiliations:** 1 Department of Applied Clinical Sciences and Biotechnology, Neuroradiology Unit, University of L'Aquila, L’Aquila, Italy; 2 Department of Applied Clinical Sciences and Biotechnology, Radiology Unit, University of L'Aquila, L’Aquila, Italy; 3 Department of Applied Clinical Sciences and Biotechnology, Radiotherapy and Cardiac Unit, University of L'Aquila, L’Aquila, Italy; University of Palermo, ITALY

## Abstract

**Objective:**

Our aim was to provide normative data concerning superior orbital fissure area (SOFA), ocular skin and the substantia nigra (D-SS) and orbital fissure and the substantia nigra (D-SOF-S) distances by CT scan in adult Caucasian population

**Methods:**

The area of the superior orbital fissure (SOF), the distance between the ocular skin and the substantia nigra and the distance between the superior orbital fissure and the substantia nigra using CT and 3D-CT images.

**Results:**

Normative data stratified for age and gender were obtained. The data here reported show that some degree of variability in SOFA, D-SS and D-SOF-S measurements can be observed healthy Caucasian subjects. Gender stratified prediction intervals (mean +/- 2 Standard Deviations) for SOFA and D-SOF-S were 69.2 (+/-15.8) and 38.4 (+/-7.6) for male and 56.8 (+/-11.9) and 36.5 (+/-6.1) for female, respectively. Age and gender significantly impacted on D-SS values and normative data were constructed generating data stratified for these two variables. D-SS was 89.4 (+/-10.3) and 86.4 (+/-9.7) for male and female, respectively.

**Conclusions:**

Here we provide adjunctive anatomical information on specific anatomical cerebral zones. Our data may have implications for surgeons actively committed to treat pathological conditions involving these cerebral areas. Additionally, the anatomical variability found with respect to SOF and the potential different exposure of the substanzia nigra to the bright light could play a role in Parkinson’s disease as already speculated in literature.

## Introduction

The superior orbital fissure (SOF) is a small and topographically important area, which connects the middle cranial fossa with the orbit [1]. It is a critical three-dimensional space with an extremely variable shape, situated laterally and below the optic canal that lies at the apex of the orbit, bounded medially by the lesser wing of the sphenoid, inferiorly and laterally by the greater wing of the sphenoid, and superiorly by the frontal bone [2]. The SOF consists of two components: the superior-lateral part and the inferior-medial part. The superior-lateral part includes the trochlear, lacrimal, and frontal nerves, and the superior ophthalmic vein. The inferior-medial part includes the superior and inferior branches of the oculomotor nerve, the naso-ciliary nerve, the abducens nerve, the sensory and sympathetic root of the ciliary ganglion. The inferior ophthalmic vein, when present, may at times pass through the tendineous annulus of Zinn [3]. So far, the morphology and the different sizes of this complex structure have been studied in cadavers [4–6]. There is no literature about the variability in size of this area observed in population of different gender. New high-tech units now available make it possible to carry out accurate measurements of the SOF, and to obtain anatomical details of this region.

## Material and Method

### Patient selection

Eighty-four patients, aged from 25 to 90 years (mean age, 59.1), were selected among subjects referred to the Department of Radiology for the execution of a CT exam of the brain for suspected stroke, not confirmed by CT follow-up, mild concussion, headache and research of metastases, without neurological symptoms. Only patients without pathological imaging findings were recruited. The patients were divided into 4 groups according to age and gender. Group A included 16 males under 50 years (age ranging from 25 to 50); Group B included 31 males over 50 years (age ranging from 51 to 90); Group C included 8 females under 50 years (age ranging from 25 to 50); and Group D included 29 females over 50 years (age ranging from 51 to 90) (Table 1). Our study received ethical approval by the Review Board of our Institution (University Ethical Committee for the assessment of non-pharmaceutical epidemiological and observational studies, University of L’Aquila, Italy). Participants provide their written informed consent to participate in this study.

**Table 1:** Demographic data of our sample.

GROUP	AGE(Lowest and highest values)	SEX	RACE	BMI (KG/M^2^)(Mean +/- SD)	NEUROLOGICAL SYMPTOMS
GROUP A	25–50	*Male*	*Caucasian*	*20*.*7 +/- 5*.*1*	*None*
GROUP B	51–90	*Male*	*Caucasian*	*22*.*8 +/- 5*.*2*	*None*
GROUP C	25–50	*Female*	*Caucasian*	*19*.*7 +/- 3*.*9*	*None*
GROUP D	51–80	*Female*	*Caucasian*	*24*.*4 +/- 3*.*9*	*None*

### CT-technique and measurements

**Fig 1:**
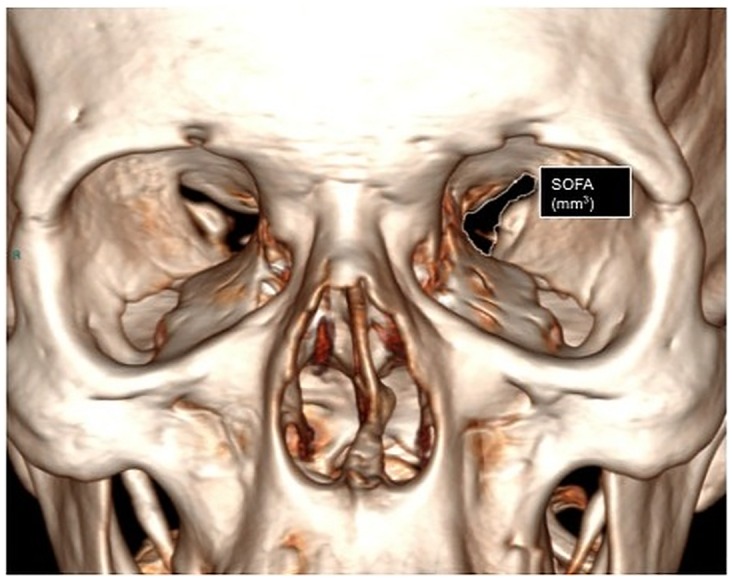
3D CT reconstruction. Measurements of the superior orbital fissure area obtained using curve measurement (SOFA).

**Fig 2:**
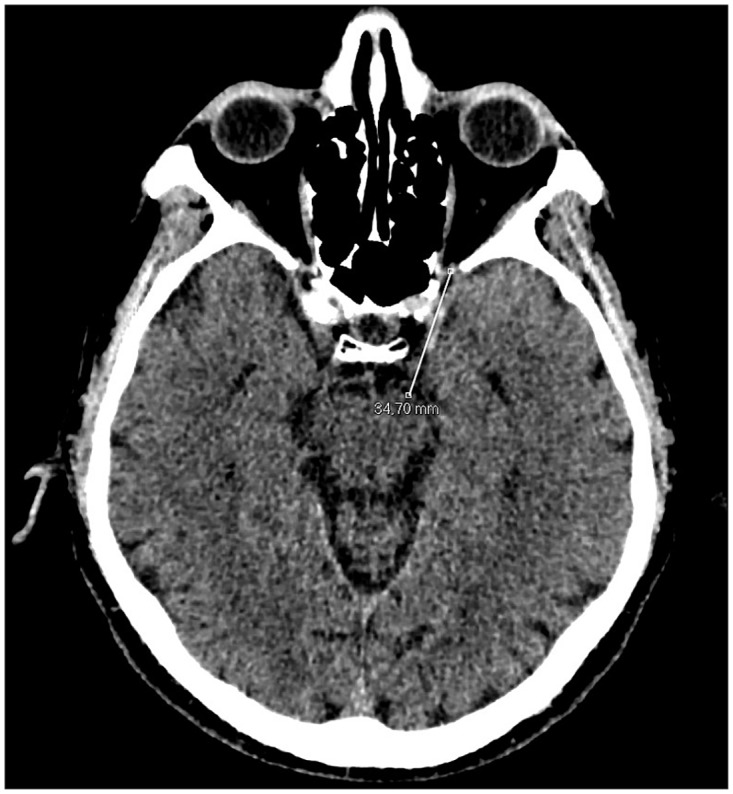
Measurements of the distances using linear system on axial CT scan. The distance between the superior orbital fissure and the substantia nigra (D-SOF-S).

**Fig 3:**
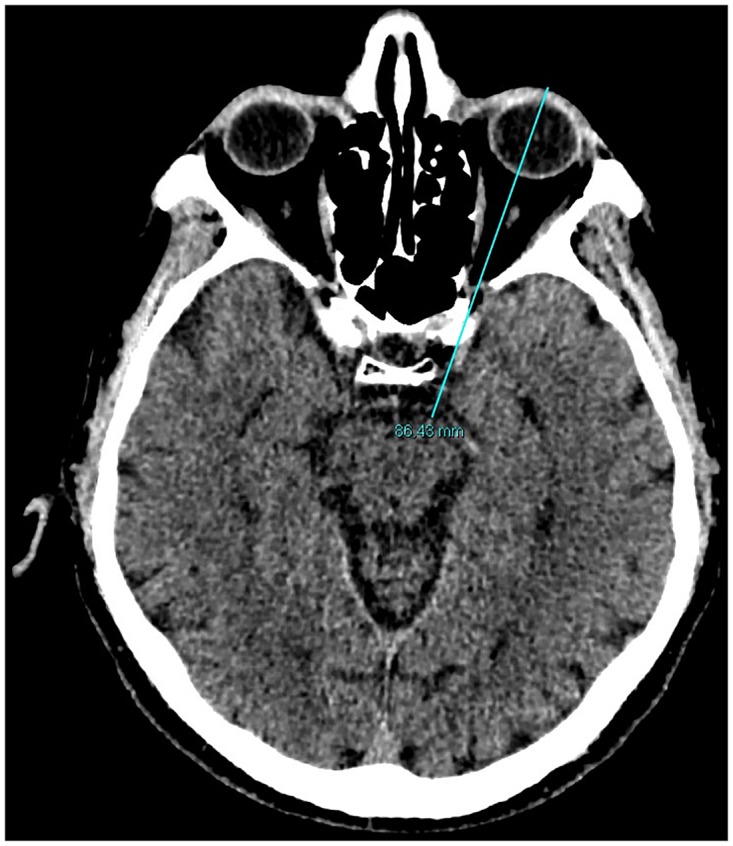
Measurements of the distances using linear system on axial CT scan. The distance between the ocular skin and the substantia nigra (D-SS).

**Fig 4:**
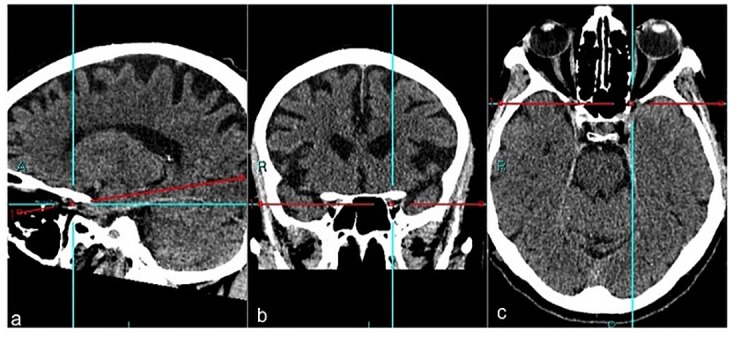
MPR reconstruction in sagittal (a), coronal (b) and axial (c) planes.

0.5 mm thickness images were acquired with a CT unit and visualized by 3 mm thickness automated reconstructions [7–9]. Superior Orbital Fissure Area (SOFA) were obtained using a curve system for the area taking into consideration the known anatomical landmarks using 3D reconstructions with 0.5 thickness following the bone margin after erasing the soft tissues (Fig 1). Linear system for the Distances between Superior Orbital Fissure and Substantia nigra (D-SOF-S) was measured on the axial/oblique planes using anatomical landmarks, that is, inferiorly and laterally to the optic canal for the superior orbital fissure, and on the anterior margin of the mesencephalon for the substantia nigra (Fig 2). The Distance between the ocular Skin and the Substantia nigra(D-SS)was measured from an external margin of the ocular bulb and the anterior margin of the mesencephalon on the same plane (Fig 3). The measurement of this distance may play an important role in the study of the outside-inside pathways of the light into the brain. This measurement was evaluated where both the superior orbital fissure and the mesencephalon could be visualized on the same plane using MPR reconstruction (MPR) (CarestreamVue PACS, version u.11.3.2.4051) (Fig 4). In this way, the measurements obtained were as reliable as possible.

The following parameters were measured:

1. The right superior orbital fissure area (RSOFA);
2. The left superior orbital fissure area (LSOFA);
3. The distance between the ocular skin and the substantia nigra (D-SS);
4. The distance between the superior orbital fissure and the substantia nigra (D-SOF-S). Two examining commissions performed the measurements. Each commission was composed by one specialist in neuroradiology and two residents, who randomly carried out the measurements, completely independently the one from the other. Concordance of results between the two commissions was evaluated with the Kendall's coefficient of concordance. Good concordance was obtained (>0.87).

### Statistical analysis

The sample size for this descriptive study was determined on the basis of the following equation: N = 4s^2^(Z_crit_)^2^/D^2^ [10], where N is the sample size of the single study group, σ is the assumed SD for the group, the Z_crit_ is a value corresponding to the desired significance criterion (alpha error 5%) and 95% confidence interval (95%CI) and D is the total width of the expected 95%CI. This equation does not depend on statistical power because it impacts on sample size only when the study design involves two or more groups [10]. Since no previous data are available in literature on the measurement of SOFA, D-SS and D-SOF-S distances by CT scan in adult Caucasian population we decided to set the limits of the 95% confidence interval (D) to no more than 1 mm, the standard deviation for the group to 2.4 mm (sigma) and the Z_crit_to 1.960. By applying these parameters the equation yields a sample size of 84 subjects. With this sample size we estimated that residual variance would attest below 30%. To test normality of continuous variables D'Agostino-Pearson test was used. Normative data were summarized by means +/- two standard deviations (+/-2SD). Means of normally distributed variables were comparing with a paired or unpaired t-test when appropriate. Not normally distributed data were log-transformed and then tested. Interrater Reliability, which determines variation between two or more raters who measure the same group of subjects, was assessed by interclass correlation coefficient (ICC). A linear regression with was used to investigate the impact of gender and age on radiological measures. ANCOVA analysis was used to generate age adjusted radiological measures. All values below the threshold of 5% in the type alpha error were considered statistically significant. All tests were performed with a dedicated statistical software (MedCalc version 13 -1993-2014 MedCalc Software bvba)

## Results

Interobserver variation between the two readers was measured with the concordance correlation coefficient (*ρ*c). The concordance correlation coefficients were all above the value of 0.912 indicating a high level of interobserver concordance. The impact of age and gender on radiological measures was evaluated by regression analysis. Gender significantly impacted on SOFA (p = 0.001), D-SS (p<0.001) and D-SOF-S (p = 0.008) whereas, age significantly impacted only on D-SS. Since a some degree of anatomical symmetry may be expected in humans, we tested the equality of R-SOFA and L-SOFA in terms of measurements. Thus, the impact of laterality on SOFA was also explored by performing a stratified analysis. As shown in Table 2 no significant difference was found between R-SOFA and L-SOFA afterpaired sample t-test.So, the two measures were combined according to the following formula: SOFA = (L-SOFA + R-SOFA)/2. When the SOFA and D-SOF-S were stratified for gender the prediction intervals with the respective reference rage (Tables 2 and 3) were 69.2 (+/-15.8) and 38.4 (+/-7.6) for male and 56.8 (+/-11.9) and 36.5 (+/-6.1) for female, respectively. Since both age and gender significantly impacted on DSS values, normative data were constructed generating data stratified for gender and adjusted for age by ANCOVA. As shown in Table 4 age adjusted DSS was 89.4 (+/-10.3) and 86.4 (+/-9.7) for male and female, respectively

**Table 2:** Normative values of superior orbital fissure area (SOFA) stratified for gender and laterality.

Gender	R-SOFA	L-SOFA	p value	SOFA
**Male (N = 47)Mean (+/- 2SD)**	68.3 (17.9)	70.1 (19.6)**	0.74*	69.2 (15.8)**
**Female (N = 37)Mean (+/- 2D)**	55.7 (13.1)	57.9 (15.1)	0.08	56.8 (11.9)**

R-SOFA = right superior orbital fissure area; L-SOFA = left superior orbital fissure area distance.

* Paired sample t-test with

** variables without normal distribution.

**Table 3:** Normative values of distance between the superior orbital fissure and the substantianigra (D-SOF-S) stratified for gender.

Gender	D-SOF-S
**Male (N = 47)Mean (+/- 2SD)**	38.4 (7.6)
**Female (N = 37)Mean (+/- 2SD)**	36.5 (6.1)

**Table 4:** Age adjusted normative values of distance between the ocular skin and the substantianigra (D-SS) stratified for gender.

Gender	D-SS
**Male (N = 47)Mean (+/- 2SD)**	89.4 (10.3)
**Female (N = 37)Mean (+/- 2SD)**	86.5 (9.7)

## Discussion

The superior orbital fissure (SOF) is a narrow bony cleft that lies at the apex of the orbit between the greater and lesser wings of the sphenoid. Through this fissure, many important structures enter the orbit from the middle cranial fossa including the third, fourth, sixth cranial nerves, and the ophthalmic branch of the fifth nerve. In addition, the superior ophthalmic vein exits the orbit to drain into the cavernous sinus via the SOF. The fissure can be divided into three anatomical regions by the annulus of Zinn (common annular tendon): The lateral, central, and inferior regions. The lateral wall of the SOF can also be divided into upper and lower segments, and the angle between them measures 144.27 degrees +/- 20.03 degrees. The advantage in collecting these data is that they provide information about the course of nerves and vasculature in the SOF [11]. There is great variation of the superior orbital fissure as described in the literature. Most of the authors distinguish 9 to 10 morphological forms of the superior orbital fissure [12–13]. Different authors reported presence of particular variants with a frequency ranging from 1.5 to 40%. Purpose of this study was to provide a method of measurement of the SOF and the distance between the ocular skin and the substantia nigra and between the superior orbital fissure and the substantia nigra.

We performed a statistical analysis of the measurements of SOF area, D-SS and D-SOF-S, by examining a sample of adult population divided into males and females, and adjusted for age. The anatomical variability of the SOF and the consequent different exposure of substantia nigra to the bright light could play a role in some pathologies such as Parkinson’s disease according to a study conducted in rats, which reports that prolonged exposure of rats to bright light induces production of neuromelanin and reduction of tyrosine hydroxylase positive neurons in the substantia nigra. Submitting a human head to CT, they identified the eye and the superior orbital fissure as a possible gateway to the ambient light to reach the midbrain, setting the stage for a more detailed study of the relationship between light exposure and Parkinson's disease, and highlighting the role of artificial light sources in this disease [14]. Through the new evaluation systems, we studied a technique to measure the area of the SOF (through 3D images) and the distance between the skin and the substantia nigra, and we extrapolated the mean values in the adult population. These data may prove useful in the study of patients with Parkinson to evaluate how the size of SOF, SS and SOF-S may represent predisposing factors for the development of the disease. We are confident that these evaluations would be helpful not only for less experienced surgeons, but also for experts to review and expand their current technique [15–17].

## Supporting Information

S1 File Database. (XLSX)
